# Development of an enzyme-linked immunosorbent assay for the detection of human calretinin in plasma and serum of mesothelioma patients

**DOI:** 10.1186/1471-2407-10-242

**Published:** 2010-05-28

**Authors:** Irina Raiko, Ingrid Sander, Daniel G Weber, Monika Raulf-Heimsoth, Adrian Gillissen, Jens Kollmeier, Arnaud Scherpereel, Thomas Brüning, Georg Johnen

**Affiliations:** 1 Institute for Prevention and Occupational Medicine of the German Social Accident Insurance of the Ruhr-University Bochum (IPA), Bürkle-de-la-Camp-Platz 1, 44789 Bochum, Germany; 2 St George Medical Centre, Robert-Koch-Hospital, Nikolai-Rumjanzew-Strasse 100, 04207 Leipzig, Germany; 3 HELIOS Clinic Emil von Behring, Respiratory Diseases Clinic Heckeshorn, Walterhöferstrasse 11, 14165 Berlin, Germany; 4 Service de Pneumologie et d'Oncologie Thoracique, Hôpital Calmette - Hospital of the University of Lille II, 59037 Lille, France

## Abstract

**Background:**

Calretinin is one of the well-established immunohistochemical markers in the diagnostics of malignant mesothelioma (MM). Its utility as a diagnostic tool in human blood, however, is scarcely investigated. The aim of this study was to develop an enzyme-linked immunosorbent assay (ELISA) for human calretinin in blood and to assess its usefulness as a potential minimally invasive diagnostic marker for MM.

**Methods:**

Initially, attempts were made to establish an assay using commercially available antibodies and to optimize it by including a biotin-streptavidin complex into the assay protocol. Subsequently, a novel ELISA based on polyclonal antibodies raised in rabbit immunized with human recombinant calretinin was developed. The assay performance in human serum and plasma (EDTA/heparin) and the influence of calcium concentrations on antibody recognition were studied. Stability of spiked-in calretinin in EDTA plasma under different storage conditions was also examined. In preliminary studies serum and plasma samples from 97 healthy volunteers, 35 asbestos-exposed workers, and 42 MM patients were analyzed.

**Results:**

The mean detection range of the new ELISA was 0.12 to 8.97 ng/ml calretinin. The assay demonstrated markedly lower background and significantly higher sensitivity compared to the initially contrived assay that used commercial antibodies. Recovery rate experiments confirmed dependence of calretinin antibody recognition on calcium concentration. Calcium adjustment is necessary for calretinin measurement in EDTA plasma. Spiked-in calretinin revealed high stability in EDTA plasma when stored at room temperature, 4°C, or after repeated freeze/thaw cycles. Median calretinin values in healthy volunteers, asbestos workers, and MM patients were 0.20, 0.33, and 0.84 ng/ml, respectively (p < 0.0001 for healthy vs. MM, p = 0.0036 for healthy vs. asbestos-exposed, p < 0.0001 for asbestos-exposed vs. MM). Median values in patients with epithelioid and biphasic MM were similar. No influence of age, gender, smoking status, or type of medium (plasma/serum) on calretinin values was found.

**Conclusions:**

The novel assay is highly sensitive and applicable to human serum and plasma. Calretinin appears to be a promising marker for the blood-based detection of MM and might complement other markers. However, further studies are required to prove its usefulness in the diagnosis of MM patients.

## Background

Malignant mesothelioma (MM) is a highly aggressive tumor of the serous membranes. MM is associated with asbestos exposure and will remain a major health problem on a worldwide scale for many decades [1,2]. Diagnosis of MM usually occurs at late stages of the disease when treatment is very difficult. There is an urgent need for markers for early diagnosis that may improve treatment options. To limit invasive diagnostic procedures, blood-based markers would be preferable. Up to now, soluble mesothelin-related peptides (SMRP) remains, despite of its low sensitivity, the best available serum marker for MM [3-5]. A marker with similar potential is the related N-ERC/mesothelin [6]. While a single tumor marker might not reach sufficient sensitivity and specificity, there is evidence that a panel of several markers could substantially improve diagnosis of cancer [7]. Moreover, reliable and non-invasive tools such as blood markers for screening of high-risk, asbestos-exposed populations are still needed.

Based on immunohistochemical results, such a potential candidate marker might be calretinin. Calretinin (calbindin 2, CALB2) is a 29 kDa calcium-binding protein, a member of the so-called EF-hand protein family frequently found in neurons [8]. It was suggested that calretinin plays a role in intracellular Ca^2+ ^homeostasis and buffering [9]. It was shown that calretinin down-regulation blocks the cell cycle and increases apoptosis in the colon adenocarcinoma cell line WiDr [10]. Recently, Henzi et al. presented evidence that calretinin plays a role in cell survival during asbestos exposure. However, its exact role in neoplasia remains unknown [11]. The presence of calretinin was also demonstrated in several other organs and tissues, among them in mesothelium [12]. Since the first evaluation of calretinin as an immunohistochemical marker of MM [13], several studies have demonstrated the significance of calretinin as a reliable marker for the diagnosis of MM, based on its high sensitivity (up to 100%) and specificity (up to 87.5%) in tumor tissues [14-17].

The relevance of calretinin as a potential blood marker for minimally invasive diagnostics of MM has not yet been investigated. Schwaller et al. described calretinin detection in human serum from cancer patients (e.g., ovarian, breast, lung), using a sandwich ELISA [18,19]. However, no sera from patients with MM were analyzed in this study and, to our best knowledge, no further attempts to determine calretinin in serum or plasma were undertaken.

Thus, the purpose of this study was to develop and optimize a novel polyclonal antibody-based ELISA for human calretinin, to test its application in human serum and plasma, and to perform a first assessment of its potential usefulness as a blood test for the diagnostic of MM.

## Methods

### Human serum and plasma samples

Serum (n = 65) or EDTA plasma samples (n = 32) were collected from 97 healthy volunteers including 54 men (mean age 45 years; range 25-76 years) and 43 women (mean age 41 years; range 25-74 years), and stored at -80°C until use. In addition to serum samples five volunteers also provided EDTA and heparin plasma samples. The 97 volunteers were recruited from employees and former employees of the IPA and the associated clinic and, based on routine occupational-medical examinations, were determined to be apparently healthy. Plasma (n = 10) and serum (n = 25) samples of one female and 34 male workers formerly exposed to asbestos and without evidence of neoplasia (mean age 69, range 55-83 years) were obtained at the IPA and the participating clinics and study centers. The asbestos-exposed group comprised persons without and with benign diseases like pleural plaques or asbestosis, representative of a group of high-risk subjects. Serum and plasma samples (n = 27 and 15, respectively) from 31 men and 11 women with MM (mean age 67 years, range 35-85 years) were analyzed as well. Samples of MM patients were either purchased from conbio (Kiel, Germany) or obtained from the French and German clinics and study centers associated with the study. Histology was known for 32 of the MM cases, with 19 epithelioid, 1 sarcomatoid, and 12 biphasic tumors. Blood samples of MM patients were collected before any anti-tumor treatment. All participants provided written informed consent and permission for the study was obtained from the ethics committee of the Ruhr-University Bochum (reference number 3217-08) or of the Hospital of the University of Lille II, France.

### Preparation of anti-calretinin antibodies

For the immunization of a rabbit 0.4 mg human recombinant calretinin (Swant, Belinzona, Switzerland) in TiterMax™ Gold as an adjuvant was injected. In intervals of five to six weeks, the rabbit was boostered twice with 0.4 mg calretinin emulsified with TiterMax™ Gold and was bled four weeks after each boost to check the titer by analysis of the serum. Four months after primary immunization the final antiserum was obtained.

The rabbit serum containing polyclonal anti-calretinin antibodies was loaded via FPLC (Pharmacia, Uppsala, Sweden) on a protein G column (GE Healthcare, Munich, Germany), washed with phosphate-buffer (20 mM, pH 7) and eluted with glycine-HCl-buffer (pH 2.8). Eluted fractions were neutralized directly with 1/10 volume 1 M Tris-HCl-buffer (pH 9), pooled and dialyzed. Protein concentration determined by BSA protein assay (Bio-Rad Laboratories GmbH, Munich, Germany) was 0.9 mg/ml. Aliquots of the purified antibodies were stored at -80°C.

The purified polyclonal anti-calretinin antibodies were biotinylated by mixing with a 33-fold molar excess of biotin-N-hydroxysuccinimid-ester (Roche Diagnostics GmbH, Mannheim, Germany) dissolved in dimethylsulfoxid and incubated under continuous agitation for 4 h at room temperature (RT). The biotinylated antibodies were dialyzed extensively and stored in aliquots at -80°C.

### Development of a two-site polyclonal antibody-based ELISA

Assay variant 1 was based on the calretinin sandwich ELISA described previously by Schierle et al. with several modifications [18]. Microtiter plates (MaxiSorp, Nunc, Denmark) were coated with goat polyclonal antibodies to calretinin (CG1; Swant, Belinzona, Switzerland) diluted 1:200 in 0.1 M carbonate/bicarbonate buffer, pH 9.6 (0.5 μg/well), and incubated overnight at 4°C. The plates were blocked with 1.5% (w/v) casein in PBS (200 μl/well) for 120 min at RT (all incubations at RT were carried out under shaking) and then washed three times with PBST (PBS containing 0.05% Tween 20). Human purified recombinant calretinin (Swant) was used as standard. A stock solution (200 μg/ml) was serially diluted in PBST to give calretinin amounts from 2000 to 0.061 ng/ml. After 90 min incubation at RT followed by washing, rabbit polyclonal antibodies to calretinin (7699/4; Swant), diluted 1:1000 with PBST (0.1 μg/well), were added and plates were incubated for another 90 min. Then, goat anti-rabbit IgG peroxidase conjugate (Sigma, Saint Louis, MO, USA), diluted 1:10000 in PBST, was added to the washed plates. After 90 min incubation and washing 100 μl/well of Enhanced K-Blue TMB Substrate (Neogen Corporation, Lexington, KY, USA) was added. The color development was stopped by the addition of 100 μl 1 M sulphuric acid per well and absorbance was measured at 450 nm using an automated plate reader (Molecular Devices, Sunnyvale, CA, USA).

In order to improve the assay's sensitivity, other modifications were applied. In assay variant 2 rabbit polyclonal antibodies to calretinin were used as capture antibody (dilution 1:300 in carbonate/bicarbonate buffer, pH 9.6). The plates were incubated overnight at 4°C, blocked for 120 min at RT and washed three times. Serially diluted calretinin standard (concentrations from 50 to 0.391 ng/ml) was added and plates were incubated overnight at 4°C. After washing the plates were incubated for 90 min with 1:1000 diluted goat polyclonal antibodies and washed again. 60 min after addition of 1:20000 diluted rabbit anti-goat IgG-biotin conjugate (Sigma, Saint Louis, MO, USA) the plates were washed three times and incubated for another 60 min with horseradish-peroxidase-streptavidin conjugate (dilution 1:20000, Fitzgerald Industries International, Concord, MA, USA). After final washing 100 μl/well of ABTS substrate solution (2.2'-azino-bis(3-ethyl-benzthiazoline-6-sulfonic acid), Sigma, Taufkirchen, Germany) were added. The substrate was activated with H_2_O_2 _according to the manufacturer's instructions. The enzyme reaction was stopped with 100 μl/well 0.32% NaF and absorbance was measured at 414 nm.

### Novel sandwich ELISA using self-developed polyclonal antibodies to calretinin

Assay variant 3 was generated with self-developed purified and biotinylated rabbit polyclonal antibodies to human recombinant calretinin. Microtiter plates were coated with purified polyclonal antibodies (100 μl/well, dilution 1:1500; 0.6 μg/ml; 100 mM carbonate/bicarbonate buffer, pH 9.6) and allowed to adhere overnight at 4°C. They were then blocked with 1.5% casein, washed and incubated with dilutions of the standard and samples for 1 h at RT. A stock solution of human purified recombinant calretinin was diluted in PBST to give standard concentrations between 10 and 0.08 ng/ml. Next, plates were washed three times and incubated for 1 h with biotinylated polyclonal antibodies (100 μl/well, dilution 1:5000; 0.18 μg/ml). After washing 100 μl/well of 1:20000 diluted horseradish-peroxidase-streptavidin conjugate (Fitzgerald Industries International, Concord, MA, USA) was added. One hour later plates were washed and 100 μl with H_2_O_2_-activated ABTS substrate solution was added to each well. The enzyme reaction was stopped by addition of 100 μl 0.32% NaF and absorbance was read at 414 nm.

The dose-response curves for standards were obtained by 4-parameter curve fitting using SoftMax Pro 4.7.1 from Molecular Devices. The lower detection limit of the assay was defined by adding 0.1 OD units (rounded 8-fold mean of the standard deviation of background values from eight plates) to the background value of each plate. Calretinin amounts above that value were considered detectable.

### Calretinin recovery rate experiments

Calretinin recovery rates were studied in serum, EDTA plasma, and heparin plasma from healthy individuals. Calretinin concentrations in all these samples were below the detection limit and were called calretinin-negative. In these experiments recovery rates were examined in the presence of various calcium concentrations from 2.5 to 10 mM in dilution buffer (TBST: Tris-buffered saline, pH 7.4 containing 0.05% Tween 20). In addition, the influence of various added calretinin concentrations on its recovery rate in plasma or serum was also studied. Prior to addition of calcium to EDTA plasma, heparin was added to a final concentration of 25 U/ml to avoid potential coagulation after calcium adjustment.

Stability of spiked-in calretinin was studied in EDTA plasma samples from five healthy calretinin-negative individuals. Calretinin (5 ng/ml final concentration) and heparin (25 U/ml plasma) were spiked into each plasma aliquot. Aliquots were handled in four different ways: 5 days at RT, 5 days at 4°C, 5 days at -80°C and 10 freeze/thaw cycles within 5 days. Afterwards, they were diluted 1:5 with 5 mM CaCl_2 _in dilution buffer (TBST) and the calretinin recovery rate was measured.

### Statistical analysis

Nonparametric Mann Whitney's U test and Kruskal-Wallis test for data comparison were performed with GraphPad Prism (GraphPad Software Inc., San Diego, CA USA). P < 0.05 was considered statistically significant. Pearson's correlation coefficient was calculated with SAS (SAS Institute Inc., Cary, North Carolina, USA, Version 9.2) to determine the correlation between age and calretinin values. The graphs were created with GraphPad Prism and SigmaPlot 8 (Systat Software Inc., San Jose, CA, USA). Values below standard range of the assay were set to a fixed value based on the four-parameter fit of the standard curve of each run.

## Results

**Figure 1:**
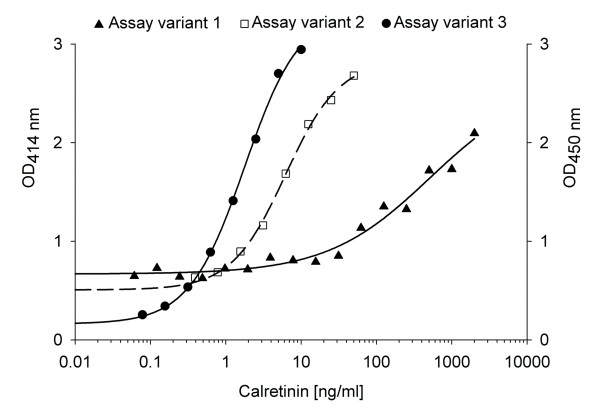
**Standard curves of calretinin enzyme-linked immunosorbent assay (ELISA) systems**. Assay variant 1: Capture and detection antibodies based on Schierle et al. [18]. Assay variant 2: Modification of variant 1 with inversion of capture and detection antibodies and amplification by a biotin-streptavidin complex. Assay variant 3: Sandwich ELISA with novel polyclonal anti-calretinin rabbit IgG antibody for capturing and detection (biotinylated).

Initially, we have used commercially available antibodies to establish a new calretinin assay. This assay was based on the only published calretinin ELISA, which was developed by Schierle et al. [19]. Goat anti-calretinin was used as capture antibody and rabbit anti-calretinin as detection antibody. This assay had a high background and a lower detection limit of 5.41 ng/ml (Fig. 1, variant 1). All attempts to improve its sensitivity and to reduce the background failed. An inversion of capture and detection antibodies (rabbit antibodies for capturing and goat antibodies for detection) had no significant effect either (data not shown). Furthermore, a biotin-streptavidin complex was included in the assay protocol. Although the background was lower than in assay variant 1, it still remained high. The lower detection limit of 0.48 ng/ml was almost tenfold lower than that of variant 1 indicating an improved sensitivity of variant 2 (Fig. 1, variant 2).

Finally, human recombinant calretinin was used to raise polyclonal anti-calretinin antibodies in rabbit. These antibodies were purified and utilized for calretinin capturing. One part of the purified antibodies was biotinylated and used for calretinin detection. Various concentrations of antibodies were tested to yield an optimized assay protocol. A representative standard curve is shown in Fig. 1 (assay variant 3). The novel ELISA demonstrated noticeably lower background and higher sensitivity than variants 1 and 2. Its lower detection limit of 0.12 ± 0.05 ng/ml (n = 11) was approximately 46-fold and 4-fold lower compared with variants 1 and 2, respectively. Also, the concentrations of the antibodies were considerably lower than those in variants 1 and 2. The optimized antibody dilutions of capture and detection antibodies were 1:1500 and 1:5000, respectively (0.6 μg/ml and 0.18 μg/ml, respectively). In this combination the assay had a mean detection range of 0.12 to 8.97 ng/ml (n = 11).

**Figure 2:**
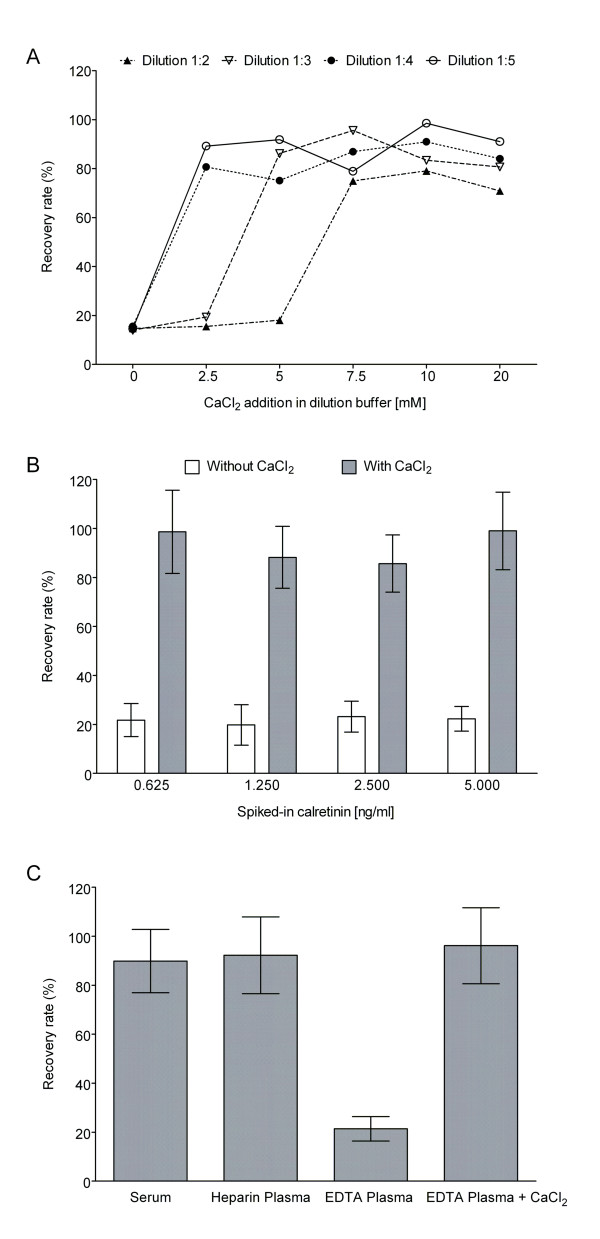
**Calretinin recovery experiments**. (A) Influence of EDTA plasma dilution and calcium concentration on calretinin recovery rate (5 ng/ml spiked-in calretinin). (B) Recovery rates of various spiked-in calretinin concentrations in EDTA plasma (n = 9). Plasma dilution 1:5, 5 mM CaCl_2 _in dilution buffer. (C) Calretinin recovery rates in serum, EDTA, and heparin plasma (n = 11). 5 ng/ml spiked-in calretinin, dilution 1:5, 5 mM CaCl_2 _in dilution buffer.

Calretinin recovery rates were studied initially in EDTA plasma of five healthy volunteers using assay variant 3. All samples revealed no measurable calretinin in preliminary examinations and were regarded as calretinin-negative. As calretinin is a Ca^2+^-binding protein its antibody recognition depends on Ca^2+ ^in the medium [20-22]. Therefore, the influence of various Ca^2+ ^concentrations in EDTA plasma on its recovery rate was studied. In addition, the influence of plasma dilutions was analyzed. In these experiments a pool of calretinin-negative EDTA plasmas was diluted 1:2, 1:3, 1:4, and 1:5 either with TBST or with TBST containing increasing concentrations of CaCl_2_. The recovery rate of 5 ng/ml calretinin was measured for each combination of Ca^2+ ^concentration and plasma dilution. As shown in Fig. 2A, without calcium addition all plasma dilutions exhibited calretinin recovery rates of just 15%. To reach a maximal recovery rate in 1:2 diluted plasma 7.5 mM CaCl_2_, in 1:3 diluted plasma 5 mM CaCl_2_, and in 1:4 and 1:5 diluted plasma 2.5 mM CaCl_2 _were necessary. Recovery rates of calretinin were slightly better in higher diluted plasmas. As a consequence of these results, most of the following experiments were performed in 1:5 diluted plasma or serum samples and 5 mM CaCl_2 _was considered to be a sufficient concentration for calcium adjustment in EDTA plasma. Additionally, the influence of different spiked-in calretinin concentrations on its recovery rate was analyzed. As shown in Fig. 2B, the spiked-in calretinin concentrations from 0.625 to 5 ng/ml revealed almost the same recovery rates, about 20-23% without calcium and about 86-99% with calcium adjustment. An improvement of the recovery rate in EDTA-plasma after calcium adjustment was observed also with assay variant 2 (data not shown).

In order to examine the influence of different media on calretinin antibody recognition, recovery rates were studied in calretinin-negative sera and plasma samples (EDTA and heparin) from the same healthy individuals. Samples were diluted 1:5 either with TBST or with 5 mM CaCl_2 _in TBST and 5 ng/ml calretinin was spiked-in to each sample. The resulting recovery rates are shown in Fig. 2C. They were 89.9 ± 12.9% and 92.2 ± 15.7% (n = 11) in serum and heparin plasma, respectively. Similar results were obtained with lower added calretinin concentrations (0.625, 1.25, and 2.5 ng/ml). Calcium addition revealed no influence on the antibody recognition of calretinin in serum and heparin plasma (data not shown). Like in the experiments with EDTA plasma dilutions, recovery rates in EDTA plasma samples without calcium addition were low (21.3 ± 5.0%). In EDTA plasma resupplied with calcium, however, they were comparable with those in serum and heparin plasma (96.1 ± 15.5%).

Spiked-in calretinin revealed a high stability when stored for 5 days at -80°C, 4°C, or even RT. Likewise, up to 10 freeze/thaw cycles had no significant influence on its recovery rate. The measured values of calretinin were from 4.9 to 5.1 ng/ml and its recovery rates were about 100% (data not shown).

**Figure 3:**
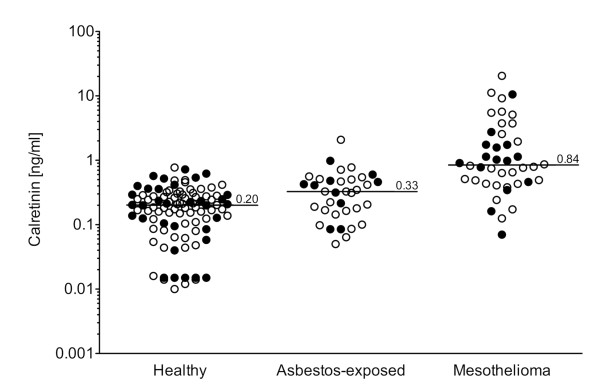
**Calretinin levels in blood samples**. Individual values for calretinin in healthy subjects (n = 97), persons with previous asbestos exposure (n = 35), and patients with malignant mesothelioma (n = 42). Open circles represent serum, filled circles plasma samples. Bars indicate median marker levels. Differences between all groups were significant (p < 0.0001 for healthy vs. MM, p = 0.0036 for healthy vs. asbestos-exposed, p < 0.0001 for asbestos-exposed vs. MM).

**Figure 4:**
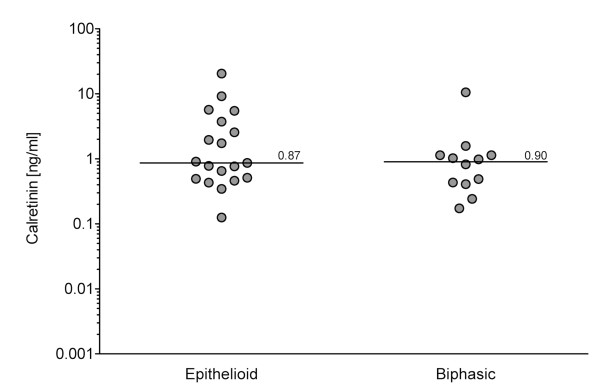
**Calretinin levels and histological subtypes**. Values of calretinin in blood samples of patients with epithelioid (n = 19) and biphasic MM (n = 12). Bars indicate median marker levels. The difference between the two subtypes was not significant (p = 0.41).

In a preliminary assessment of calretinin as a possible tumor marker its concentrations were measured in 1:2 to 1:4 diluted sera from healthy subjects (n = 65) and from patients with diagnosed MM (n = 27) as well as in 1:5 diluted EDTA plasmas (32 healthy subjects and 15 patients with MM). As shown in Fig. 3, the median values of calretinin in serum and plasma of MM patients were significantly higher than in blood samples from healthy controls (0.84 vs. 0.20 ng/ml; p < 0.0001) or from asbestos-exposed workers (0.84 vs. 0.33 ng/ml; p < 0.0001). There was also a significant difference between calretinin blood levels in asbestos-exposed workers *versus *healthy controls (0.33 vs. 0.20 ng/ml, respectively; p = 0.0036). Regarding the histological MM subtype, patients with epithelioid MM did not exhibit significantly different median serum levels of calretinin than patients with biphasic MM (0.87 vs. 0.90 ng/ml, respectively; p = 0.41) (Fig. 4). Because we had only one sarcomatoid MM in our collective, a meaningful statistical analysis for this subtype was not possible. Median calretinin values in serum vs. plasma revealed no significant differences within the group of healthy controls (0.19 vs. 0.21 ng/ml, respectively; p = 0.69), asbestos-exposed workers (0.31 vs. 0.42 ng/ml, respectively; p = 0.55), or MM patients (0.77 vs. 1.02 ng/ml, respectively; p = 0.96). There was no significant difference in the median blood calretinin value in the healthy subjects group, depending on their age (Pearson's correlation coefficient, r = -0.11; p = 0.27), their sex (men vs. women: 0.21 vs. 0.20 ng/ml; p = 0.56), or their smoking status (smokers (n = 11), ex-smokers (n = 11), and non-smokers (n = 45): 0.23, 0.23, and 0.21 ng/ml respectively; p = 0.83).

## Discussion

Calretinin is one of the best and most frequently used markers in the immunohistochemical diagnosis of MM [17,23]. Its value as a blood-based tumor marker in MM remains to be proven. A minimally invasive blood marker would greatly improve diagnosis and early detection of MM. Schwaller et al. detected calretinin and its splice form calretinin-22k in sera from patients with tumors of various localizations as well as from healthy persons [19]. Although the calretinin values in the patient group were considerably higher than in the control group, the relatively low number of samples in each category did not allow for an association with the pathological condition. To our knowledge, no other studies to assess soluble calretinin in human blood have been performed yet.

To develop a novel sandwich ELISA, we have used polyclonal antibodies raised in rabbit after injection of human recombinant calretinin. Compared to assay variants 1 and 2 with commercial antibodies, the novel assay is reproducible, provides higher sensitivity (about 46 and 4 times higher than those of variants 1 and 2, respectively), has noticeably lower background, and exhibits a steeply rising calibration curve. In the assay variants 1 and 2 capture and detection antibodies are polyclonal and were raised in different species (rabbit and goat, respectively). To facilitate a final enzyme reaction in these assay variants, a labeled secondary antibody against the detection antibody was used (e.g., goat anti-rabbit IgG peroxidase conjugate in variant 1). Although this secondary antibody was raised in the same species as the capture one (goat in variant 1), it revealed some non-specific binding to the latter (data not shown). The high background observed in the assay variants 1 and 2 was partially caused by this non-specific binding. In the novel ELISA we have used biotinylated anti-calretinin as a detection antibody, thereby a labeled secondary detection antibody was dispensable. The background of this assay variant is significantly lower, supporting our explanation of the high background in assay variants 1 and 2. Furthermore, the novel assay has fewer steps and requires less time. In addition, considerably lower concentrations of capture and detection antibodies are required for its performance (0.6 μg/ml and 0.18 μg/ml compared to 5 μg/ml and 1 μg/ml for variant 1, respectively) and therefore it is less expensive.

The choice of appropriate sample type (serum or plasma) and anticoagulant (EDTA, heparin, or citrate) is of great importance for the practical use of assays. Until now calretinin concentration in human blood was determined only in serum samples [19]. In the present study we investigated for the first time calretinin values in EDTA and heparin plasma as well as in serum, using recovery rate experiments.

In our initial studies we have observed very low recovery rates of spiked-in calretinin in EDTA plasma (15-20%). They were clearly improved after addition of CaCl_2 _to the dilution buffer. Similar effects were described for the ELISA-based detection of S100A12 in EDTA plasma, another Ca^2+^-binding protein of the EF-hand family [24]. It is known that calretinin antibody recognition depends on its Ca^2+^-binding status and it was experimentally confirmed that Ca^2+ ^induces conformational changes in the calretinin molecule that likely promote antibody recognition [20-22]. These findings explain the initially low recovery rates observed in our experiments, because EDTA strongly binds calcium so that the remaining concentration in plasma is not sufficient to provide appropriate conformational changes necessary for optimal antibody recognition. Subsequently, we found a Ca^2+ ^concentration in the dilution buffer of 5 mM in combination with a plasma dilution of 1:5 to be optimal. Also, due to the lower viscosity of diluted plasma antibody recognition is less impeded and assay reproducibility is thus improved.

Despite rules for proper tissue banking, in reality samples might not always be handled according to standard operating procedures, leading to altered marker levels [25]. Our present study indicates that calretinin concentration in plasma is not altered after repeated freeze/thaw cycles or during the storage at RT or 4°C for at least five days. These results suggest that calretinin might be a stable marker applicable in field studies.

Using our sandwich ELISA, we determined calretinin levels in serum and EDTA plasma from patients with MM, asbestos-exposed persons (with and without benign lung or pleural diseases), and healthy controls. Significantly higher values were observed in MM samples compared to samples of healthy volunteers or asbestos-exposed persons without malignancies. This result - after confirmation in a larger group of patients - may suggest blood calretinin as a soluble marker for the (early) diagnosis of MM.

Current MM markers such as soluble mesothelin (SMRP) mainly detect epithelioid MM, while biphasic and especially sarcomatoid MM are frequently missed. Based on immunohistochemistry, calretinin is known to be detected in epithelioid MM but can also be found in other MM subtypes [13,17]. Our results indicate that this finding - at least for epithelioid and biphasic MM - could be extended to the detection of this marker in blood samples.

On the basis of our initial results we suggest that the measurement of calretinin in human serum and plasma might be a useful marker for the diagnosis of MM, alone or combined with other markers such as soluble mesothelin. However, these results are based on relatively small numbers and further studies on more patients, including subgroups of subjects with other tumors and non-malignant lung or pleural diseases, are needed to confirm our initial data. Such a study is presently ongoing. In addition, to validate a new marker for early detection of cancer the most suitable design is that of a prospective study. Currently, we are establishing a large cohort of patients with benign asbestos-related diseases to evaluate calretinin and other potential markers within a longitudinal study.

## Conclusions

We have developed a novel and sensitive ELISA for the detection of calretinin in serum, heparin plasma, or EDTA plasma augmented with calcium. No significant differences between calretinin recovery rates in serum or plasma were detected. Also, high calretinin stability was observed under several storage conditions as well as after repeated freeze/thaw cycles. Calretinin levels were significantly increased in MM patients, suggesting that it might be suitable - possibly in combination with other markers like mesothelin - for the blood-based detection of MM. These factors provide a promising basis for the validation of calretinin as a biomarker for MM in larger case-control as well as prospective studies.

## Competing interests

The authors declare that they have no competing interests.

## Authors' contributions

IR carried out the immunoassays, performed the statistical analysis, and drafted the manuscript. IS participated in the design of the assays and helped to draft the manuscript. DGW participated in the design and coordination of the study and helped to draft the manuscript. MRH helped with the assay design and the draft of the manuscript. JK participated in the coordination of the study and helped with the draft. AG participated in the coordination of the study and helped with the draft of the manuscript. AS participated in study design and patient recruitment and helped to draft the manuscript. TB participated in the study design and coordination. GJ conceived of the study, participated in its design and coordination, and helped to draft the manuscript. All authors read and approved the final manuscript.

## Pre-publication history

The pre-publication history for this paper can be accessed here:

http://www.biomedcentral.com/1471-2407/10/242/prepub
